# The International Patient Decision Aid Standards (IPDAS) Collaboration: Evidence Update 2.0

**DOI:** 10.1177/0272989X211035681

**Published:** 2021-08-20

**Authors:** Dawn Stacey, Robert J. Volk, for the IPDAS Evidence Update Leads (Hilary Bekker, Karina Dahl Steffensen, Tammy C. Hoffmann, Kirsten McCaffery, Rachel Thompson, Richard Thomson, Lyndal Trevena, Trudy van der Weijden, and Holly Witteman)

**Affiliations:** School of Nursing, University of Ottawa; Ottawa Hospital Research Institute, Ottawa, ON, Canada; Department of Health Services Research, the University of Texas MD Anderson Cancer Center, Houston, TX, USA

Established in 2003, the International Patient Decision Aid Standards (IPDAS) Collaboration aims to enhance the quality and effectiveness of patient decision aids (PtDAs) by establishing a shared evidence-informed framework to guide developers and researchers in their development, content, evaluation, and implementation. The original IPDAS checklist, based on evidence syntheses, focused on components of PtDAs known to support informed, values-based reasoning and engagement with health care professionals. In this article, we present IPDAS Evidence Update 2.0. The 13 articles that make up this update provide the latest evidence on 11 core IPDAS domains: development process,^1^ providing balanced information,^2^ communicating probabilities of outcomes,^3,4^ clarifying values,^5^ using personal stories,^6^ guidance and decision coaching,^7^ disclosing conflicts of interest,^8^ health literacy,^9,10^ basing information on scientific evidence,^11^ measuring effectiveness,^12^ and implementation of PtDAs.^13^

## History of the IPDAS Collaboration

PtDAs are evidence-informed resources to guide patients in the process of making quality decisions.^14^ At a minimum, PtDAs describe the health condition or problem; make explicit the decision; provide information on options, benefits, and harms; and help patients clarify which benefits and harms matter most.^15^ Optional features in PtDAs are probabilities of outcomes of options, narratives describing patients’ experiences with making decisions, and guidance in the process of decision making. They are designed to be used as adjuncts to counseling and are often used to facilitate shared decision making between patients and their clinician. A systematic review of 105 randomized controlled trials demonstrated that compared with usual care, patients exposed to PtDAs have improved knowledge, more realistic expectations, less decisional conflict and participate more actively in making decisions.^16^ Given that few have been used in clinical practice after trials were completed,^17^ there is increasing research focused on the process used for their development, evaluation, and implementation.

Evidence was emerging in 2003 that PtDAs can affect the uptake of options.^18^ For example, there were decreased hysterectomies and fewer herniated disc surgical procedures when patients were aware of nonsurgical options to address the condition. The effect on uptake of options was judged to be positive when PtDAs were unbiased and the change addressed variations in clinical practice.^19,20^ Concurrently, there was concern that PtDAs developed without a guiding set of standards could be used to present biased information.

**Figure 1:**
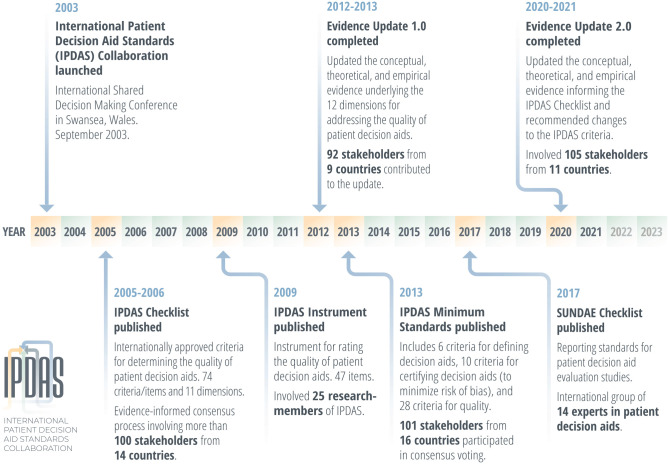
History of the International Patient Decision Aid Standards Collaboration.

In 2003, the IPDAS Collaboration was established to enhance the quality and effectiveness of PtDAs by establishing a shared evidence-informed framework for improving their content, development, evaluation, and implementation.^21^ The collaboration has been an entirely volunteer organization with no formal affiliation with a professional society, and members have produced a series of evidence-based IPDAS resources (Figure 1). IPDAS used an international consensus process to establish the first set of criteria within 12 broad domains for determining the quality of PtDAs.^19^ There was representation from 14 countries, with more than 100 participants including researchers, clinicians, patients, and policy makers. Based on equi-median ratings of 7 to 9 out of 9, the original IPDAS checklist included 74 items from 11 of the broad domains with a present/absent response scale. The only domain not included was *patient narratives*, given conflicting evidence. Next, the IPDAS instrument for measuring quality was created and validated with only 47 items described on a 4-point scale ranging from *strongly agree* to *strongly disagree*.^22^

Given the number of IPDAS items and some challenges with applying the items, IPDAS proposed a minimum set of standards for defining and certifying PtDAs.^15^ There were 127 participants from 16 countries who had some experience with PtDAs who voted on “if the criterion was not present or of low quality, there would be a risk of harmful bias and potential negative impact on patients’ decision making.” Considering the numeric and qualitative results from voters, the original IPDAS rating (1 to 9), and comments on the feasibility from those trained in using the IPDAS instrument, the expert committee proposed 6 criteria for qualifying to be defined as a PtDA, 6 criteria for certifying PtDAs, plus 4 for screening PtDAs (to minimize risk of bias), and others were described as quality criteria. In 2013, IPDAS members published the updated theoretical and empirical evidence on the 12 original broad domains, plus 1 extra team published evidence on implementation of PtDAs.^14,23^ In addition to supporting the IPDAS criteria, this update provided more detailed guidance on developing PtDAs and discussed ways of describing the quality of the evidence used to inform PtDAs (e.g., GRADE ratings) and the need to disclosure actual or potential conflict of interest, particularly for funding received from commercial for-profit entities used to develop or exclusively distribute PtDAs. The 2013 evidence update did not include changes to the IPDAS criteria at that time.

In 2016, Washington State Health Care Authority launched the first program to certify PtDAs based on the IPDAS criteria.^24^ The certification program is noteworthy because it provides a heightened level of legal protection to clinicians who use certified PtDAs with their patients.^25,26^ This program typically announces a call for PtDAs based on specific conditions (e.g., vaginal birth after caesarean, joint replacement), and certified PtDAs are announced on their website. Concurrently, the IPDAS criteria are: being used by the Norwegian Health Department for reviewing PtDAs approved for the national platform,^27^ being used for the International A to Z Inventory at the Ottawa Hospital Research Institute,^28^ formally approved in the Netherlands by national stakeholders,^29^ and they were proposed for national standards for certification of PtDAs by the National Quality Forum.^20^ The standards are also available in Japanese, Spanish, and Chinese (IPDAS website).^21^

In 2018, the IPDAS reporting guidelines workgroup published the Standards for Universal reporting of patient Decision Aid Evaluations (SUNDAE) Checklist.^30–32^ Based on the IPDAS quality dimensions and other reporting guidelines, the 26-item SUNDAE Checklist is meant to promote greater transparency and completeness of intervention studies that evaluate PtDAs.

Given the increased use of IPDAS, the rapidly growing number of clinical practice guidelines recommending PtDAs,^33^ and the wealth of new research about their use and effectiveness, the IPDAS Steering Committee identified the need for another evidence update with a specific focus on identifying recommendations for changes to the IPDAS criteria.

## Strategy for Updating the Evidence about PtDAs

In fall 2018, the IPDAS Steering Committee identified 11 team leads for each of the 12 original broad domains with 2 changes: 1) balanced information was merged with the presentation of information on options, benefits, and harms and 2) the delivery of PtDAs on the internet was merged with the implementation of PtDAs. Senior researchers were chosen based on their involvement in previous evidence updates and their research in the area of the specific domain. They were encouraged to identify co-leads from another country. Volunteers for each of the domains were recruited through the IPDAS listserv and at the 2018 Society for Medical Decision Making Shared Decision Making special interest group meeting in Montreal, Canada. Concurrently, we asked for other topics that should be included in this update.

Domain teams were tasked with drafting a proposal for the process they planned to use for updating the theoretical and empirical evidence published since the 2013 update and making recommendations of changes to the original IPDAS criteria. Teams were given examples from the 2013 update and asked to create an update of publishable quality. The proposals were reviewed by members of the IPDAS Steering Committee in spring 2019 based on the following criteria: 1) names and affiliations of working group leaders and members with representation from 2 or more countries, 2) proposal based on previous IPDAS work including definitions and original criteria, 3) proposed methods aim to synthesize the best available theoretical and empirical evidence, 4) indication in the proposal that 1 outcome of Update 2.0 is verifying and/or revising the original criteria with justification for changes, 5) timeline aims to have work completed, and 6) completed disclosures of interest.

The IPDAS Steering Committee gave careful attention to how potential conflicts of interest would be disclosed among the team leads and members. At the outset of the update, members were asked to declare direct interests where there was an opportunity for financial gains (income from grants, contract, consulting fees, scholarships, royalties, and patents) for themselves, a spouse, or dependent children. Other reportable debts, outside positions, agreements or arrangements, and gifts or travel were also disclosed. Finally, indirect interests where there was an opportunity for benefit for a third party closely associated with the member were also disclosed. Declarations of interest were regathered at the time of release of the updates from all members.

## Current Evidence Update

The update involved 105 unique participants from 11 countries. While the IPDAS Steering Committee did not determine any conflicts that rose to the point of disqualifying a member from participating in the update, promoting transparency is an ongoing priority of the collaboration. The articles in the IPDAS Update 2.0 series reflect the 11 broad domains.^1–13^ Two of the broad domains published 2 articles: communicating probabilities about outcomes^3,4^ and health literacy.^9,10^ Other topics suggested for this update were theories and mechanisms, training in shared decision making, application of shared decision making in support of chronic conditions, whether or not to provide probabilities, and targeting specific disadvantaged populations. Given IPDAS’s mandate is focused on PtDAs, we excluded suggestions more broadly focused on shared decision making and asked teams to report on updated theories and mechanisms. The other 2 suggestions were assumed by the communicating probabilities team and the health literacy team.

## Toward Updating the Standards

IPDAS is commonly used to inform the development and evaluation of PtDAs. Evidence continues to support the minimal criteria. Although a few new criteria were proposed by authors of articles in this update, the IPDAS Steering Committee will engage in a broader consensus process before changes will be made to the IPDAS criteria. Ongoing updates such as these are a vital part of maintaining the IPDAS criteria as responsive to changes in emerging evidence and relevant to PtDA development, content, evaluation, and implementation.
